# Editorial: Bioengineering and biotechnology approaches in cardiovascular sciences, volume III

**DOI:** 10.3389/fbioe.2026.1893954

**Published:** 2026-06-16

**Authors:** Thanh Nguyen, Issei Komuro, Jianyi Zhang

**Affiliations:** 1 Department of Biomedical Engineering, The University of Alabama at Birmingham, Birmingham, AL, United States; 2 Department of Frontier Cardiovascular Science, The University of Tokyo Graduate School of Medicine, Tokyo, Japan; 3 International University of Health and Welfare, Tokyo, Japan; 4 Department of Medicine, Division of Cardiovascular Disease, School of Medicine, University of Alabama at Birmingham, Birmingham, AL, United States

**Keywords:** cardiac tissue maturation, cardiovascular diseases, engineered cardiac tissues, engineered tissue model, heart failure

Heart failure remains a leading cause of mortality worldwide, with only a 50% of heart failure patients surviving within 5 years (Zhang et al., 2025). In heart failure, the heart muscle loses its contractility; therefore, new therapeutic approaches focus on replacing the dysfunctional muscle with engineered, functional cardiac tissue or remuscularizing the endogenous heart muscle (Salar Amoli et al., 2024).

After more than 20 years of development, cardiac tissue engineering has become a mature field, leading to 10 cell-based clinical trials for heart failure worldwide (Zhang et al., 2025). Therefore, ongoing development in cardiac tissue engineering has been turning toward optimizing the maturity and contractile performance of the engineered tissue. This could be achieved by modulating a cell adhesion regulator, such as Laminin (Casarella et al.), or a gap junction regulator, such as Connexin 43 (Takada et al.). New, cross-platform, and more efficient approaches to assess engineered cardiac tissue are also proposed, such as using the engineered tissue’s transcriptomic profile (Chanthra et al.).

In addition to its therapeutic potential, engineered cardiovascular tissue could be used for modeling cardiovascular diseases and drug testing. For example, human-induced pluripotent stem cell-derived cardiomyocytes are widely used to study the characteristics of Dilated Cardiomyopathy, Hypertrophic Cardiomyopathy, Long QT Syndrome, Brugada Syndrome, Short QT syndrome, Myocardial Infarction, and Cardiotoxicity (Moriwaki et al.). Engineered heart valves are used in modeling and treating Prosthetic Heart Valves (Evangelista et al.). The human umbilical cords and placentas, collected during clinical cesarean section procedures, could be used to model Extracorporeal Membrane Oxygenation cannulation in artificial placenta technology (Hoyos Banchon et al.). These strategies offer alternative approaches to *in vivo* studies and could potentially reduce the time and cost of studying cardiovascular diseases while maintaining key disease-specific pathological phenotypes.

In summary, advances in engineering cardiac tissue are important for *in vitro* disease modeling and drug screening, as well as for various clinical therapies. On the other hand, recent mechanistic findings in endogenous cardiac regeneration (Olson, 2024) may offer a new direction for developing new engineering approaches to treat cardiovascular diseases.
